# Women and Their Victims

**Published:** 1888-12-01

**Authors:** 


### Women and their Victims.

Kathleen writes:—The Rev. F. O. Morris, Rector of Nunburnholme, Yorkshire, so well-known as the friend and historian of all "feathered folk," has been good enough to send me a very kind letter expressing approval of some remarks of mine in a column of "Tea Table Talk" which I have the honour to contribute weekly to *The Leeds Mercury Supplement*. These remarks referred to the question of the immense amount of bird-slaughter involved by the present craze for wearing birds as part of feminine adornment. Mr. Morris has also sent me The Hospital of November 10th, containing a letter from "A Woman Who Does Not Wear Birds," and he has asked me to reply to it, as he has, he thinks, exhausted all he has to say on the subject of the poor persecuted bird in his various books and pamphlets. I have not the pleasure of knowing Mr. Morris, but I hope he will allow me, as one of his sincerest admirers, to express a hope that he is wrong in so thinking.

As regards the letter of "A Woman Who Does Not Wear Birds," I think that your pithy editorial note embodies all that need be said, as far as *she* is concerned, although it concerns me to add, as a traveller in all tropical countries, that the destruction of bird-life by what she, with some slight verbal confusion, terms "serpents or other wild animals probably," approximates in no appreciable degree to their slaughter for the purposes of trade. Further than this, I hope you will allow me to point out that in my article referring to the subject, which Mr. Morris has, I believe, sent you, I mentioned with genuine disgust and horror that the birds which crammed the milliners' windows were, with very few exceptions, British birds, such as robins, magpies, bullfinches, goldfinches, sparrows, kingfishers, jays, and various sea-birds. I think I may fairly conclude that these birds had been slaughtered solely for trade purposes, since in our isles they enjoy a comparative immunity from the attacks of "serpents and other wild animals probably."

A correspondent has also sent me a copy of the *Englishwoman's Review*, in which a lady, signing herself "A Chattel," and mixing up this question of bird-murder and women's suffrage in a curiously-ingenious manner, goes out of her way to prove that men, and *not* women, are to blame for this fashion of wearing birds as adornments, and she adds that "women of the wealthier classes do not wear birds in their headgear." I altogether dispute this statement. The women of the wealthier classes are those who, as a rule, can afford to have caprices and "set the fashion." Our poorer sisters merely imitate them. "A Chattel" adds that "men kill for killing's sake," and says that "if any man who feels a sufficient curiosity on the subject will ask in a bonnet shop what is the reason of the reappearance of birds as trimmings, he will be told that it is due entirely to the men who gain their living by it, and that there is no other trade they like so well!" Then she quotes the well-worn case of "the peer who maims unfortunate pigeons at Hurlingham," but she possibly finds it inconvenient to remember the example of the peeress who, by her presence at these tournaments of doves, adds encouragement to their slaughter. I am ashamed to take up your space by replying to such inconsequent arguments as these, but when I remember that the preparation and mounting of birds to wear is mainly, if not entirely, the work of women, I may be permitted to point out that many more of my sex "gain their living" by such work than men. Further than this, when I read in fashion papers reports of this or that *grande dame's* prowess with the gun, or when my tailor sends me a circular, giving, in the same sentences as those he devotes to my riding habit, or walking costumes, detailed accounts of the newest thing in "shooting-gowns," what can I conclude but that the demand for such dresses creates the supply?

For goodness' sake don't let women try to shuffle out of this difficulty. Do let us have the courage of our opinions, and admit the fact that there never was a time in the history of women when we had more influence—more, in fact, of our own way—than the present. Let us be honest enough to own that so long as we demand birds to wear, so long will people be found to gratify our caprices. We women are essentially imitative animals, and if we were, alas! only logical also, we should recognise the force of example, and the certainty that while "the women of the wealthier classes" insist upon decorating themselves with the corpses, or parts of the corpses, of birds, so long will our poorer sisters "follow the fashion" we set them.

---

Everyone knows, I presume (writes a correspondent of the *Pall Mall Gazette*) that there is a society of ladies in London, presided over by the Princess of Wales, which aims at the abolition of the cruel and foolish fashion of wearing stuffed birds as ornaments for female attire. I had the curiosity the other day to inquire of a fashionable Bond Street vendor of ladies' hats how the principles of this society affected her trade. "I don't find that it makes much difference," replied Mdme. L——. And, indeed, nearly all the smartest hats in her window were adorned with the skins and feathers of brilliantly-coloured birds. "I suppose the ladies who belong to the society do not deal here?" I queried. "Oh yes, they do," said Madame. "I have ten or a dozen of them on my books; but when I sell one of them a hat trimmed with birds, *I have to cut the heads off;* they don't mind wings and tails, but they think it cruel to wear the heads!" Alas, poor humanity!

